# EEG Correlates of the Influence of Somatosensory Input, Expectations and Trait‐Like Bias on Pain Perception

**DOI:** 10.1002/ejp.70154

**Published:** 2025-11-07

**Authors:** Ariane Delgado‐Sanchez, Christiana Charalambous, Hannah Safi, Anthony Jones, Christopher Brown, Nelson J. Trujillo‐Barreto

**Affiliations:** ^1^ School of Psychology Manchester Metropolitan University Manchester UK; ^2^ Division of Human Communication, Development, and Hearing University of Manchester Manchester UK; ^3^ Department of Mathematics The University of Manchester Manchester UK; ^4^ Medical Physics Department, Salford Care Organisation Northern Care Alliance NHS Foundation Trust Salford UK; ^5^ Department of Electrical and Electronic Engineering The University of Manchester Manchester UK; ^6^ Institute of Population Health University of Liverpool Liverpool UK

## Abstract

**Background:**

The weighting of somatosensory input and pain expectation during pain perception is promising for pain phenotyping, with good test–retest reliability. Yet, their concurrent validity with neural and psychological variables requires further investigation.

**Objectives:**

In this cross‐sectional study, we investigated the concurrent validity of these weights with EEG source correlates of the somatosensory and expectation components during pain processing.

**Methods:**

Participants completed a cued pain paradigm, with EEG recorded during pain expectation and perception. We used Bayesian inference to estimate the participant‐specific weighting of somatosensory input, expectations and trait‐like bias, and identified sources of brain activity at different stages of the cued pain task (early anticipation, late anticipation and post‐stimulation). We correlated the estimated weights with EEG source activity across individuals.

**Results:**

As hypothesised, the weight placed on somatosensory input correlated with source activity in areas related to attention (middle frontal gyrus) and sensory processing (postcentral gyrus) during late anticipation. The expectation weight positively correlated with activity in areas related to attention (middle frontal gyrus) and semantic processing (medial temporal gyrus). We found no significant correlations between any of the weights and analgesic or hyperalgesic psychological variables (mindfulness, pain catastrophising and attachment).

**Conclusion:**

Our findings support the concurrent validity of sensory and expectation weights with related EEG source activity in pain perception, reinforcing their utility in pain phenotyping and paving the way for more personalised pain management.

**Significance:**

Our findings support the concurrent validity of sensory and expectation weights extracted through a Bayesian model. This finding supports the use of these weights for pain phenotyping.

## 1 Introduction

The relative contributions of somatosensory input and expectations to pain perception, as inferred from a Bayesian computational model of pain processing (hereafter referred to as somatosensory and expectation weights) have emerged as a promising approach for pain phenotyping (Hoskin et al. 2019). This approach could serve to identify the most influential factors on a person's pain response and therefore be used to personalise treatments. Our previous research found these weights to have good test–retest reliability (Delgado‐Sanchez et al. 2023). However, before considering their clinical application there is a need to assess whether these weights measure the intended constructs.

To do this, the usual approach is to explore the correlations from the new constructs with the scores to different psychological questionnaires that could be explored. Nevertheless, most psychological constructs currently present a valenced and confounded view of somatosensory and expectation weights. For instance, mindfulness represents attention to somatosensory input with an associated positive valence and the cognitive process of re‐evaluation of the experience incorporated in the construct (Van Dam et al. 2018). Since attention to somatosensory input (a proxy of sensory weight; Trapp and Vilares 2020) has been associated with hyperalgesia (Buhle and Wager 2010; Vlaeyen and Linton 2000) and analgesia (Johnston et al. 2012; de Bruin et al. 2020) in experimental and clinical settings, it is not possible to reliably construct a hypothesis of whether our weights would be associated with a positively or negatively balanced psychological construct.

Consequently, in this study we used the alternative approach of correlating the estimated weights with Electroencephalogram (EEG) activity during pain anticipation and pain processing as an approach for initial validation. Previous studies have identified scalp EEG signal features and source activity that correlate with the influence of sensory information and expectations. The P2 peak amplitude correlates with sensory influence (Nickel et al. 2022), whereas attention to somatosensory information is correlated with activity in the somatosensory cortex (Jones et al. 2010). Moreover, sensory influences on pain have been related to activity in brain areas related to attentional control (middle frontal gyrus [MFG], posterior cingulate cortex and inferior parietal cortex) (Brown et al. 2008), while higher reliance on expectations/cues is associated with increased activity in attentional control areas (subgenual cingulate cortex and MFG) and higher order cognition (superior frontal gyrus, inferior temporal cortex, inferior frontal gyrus and orbitofrontal cortex) (Brown et al. 2008; Watson et al. 2009). Correlations with activity in these sources would provide initial validation of the estimated weights.

Therefore, we tested the initial validity of the weight placed on somatosensory input and expectations through their correlation with EEG features during a cued pain paradigm. Participants' somatosensory input, expectations and trait‐like bias weights were computed and correlated with the magnitude of the reconstructed EEG source activity during pain anticipation and pain perception. We expected to see a positive correlation between the somatosensory weight and activity in areas associated with sensory and attentional processes. We also expected a positive correlation between expectations weight and activity in areas associated with attention and higher order cognition. We also hypothesised that both the weight placed on somatosensory input and trait‐like bias would be correlated with the magnitude of the P2 peak of the evoked Event Related Potential (ERP).

Furthermore, as mentioned previously, there is conflicting evidence regarding the analgesic/hyperalgesic effects of the somatosensory input weight (Buhle and Wager 2010; de Bruin et al. 2020; Johnston et al. 2012; Vlaeyen and Linton 2000). Therefore, as a secondary hypothesis, we tested whether the weights represent protective or vulnerability factors in correlation with pain. To do this, we correlated our weight estimates with vulnerability (pain catastrophising, attachment insecurity) and protective (mindfulness) variables shown to have an influence on both experimental and clinical pain (Harrison et al. 2019; Meredith et al. 2006; Sullivan et al. 2004).

## 2 Methods

### 2.1 Participants

Seventy right‐handed healthy participants were recruited for the experiment. A priori sample calculations (carried out with G*power; Faul et al. 2007) showed that this sample size would be sufficient to detect a one‐tailed correlation at a power of 0.8 with a medium effect size (0.33). Note that previous research similar to this one, albeit with substantial differences in methods and measured factors, has found effect sizes that range from medium to large (Brown et al. 2008; Lim et al. 2020), which we used to base our expected effect size for our sample calculation. The exclusion criteria included a history of chronic pain, the presence of substantial psychiatric history, the use of prescription medications that might influence brain functioning and a history of drug or alcohol abuse. Volunteers were reimbursed at a rate of £80 per session. The study received ethical approval from the University of Manchester's Research Ethics Committee 2 (UREC 2), and all participants gave written informed consent. Seven participants were excluded from the experiment due to high pain tolerance and/or incorrect completion of experimental procedures. The final sample consisted of 63 participants (36 females, 27 males; mean age 38.4, SD 15.79). This sample size was still sufficient to achieve the required power and effect size levels.

### 2.2 Experimental Procedure

#### 2.2.1 Psychophysics

Electrical stimulation was delivered through an in‐house built electrical stimulator (Medical Physics department, Salford Royal Hospital) that was connected to the participants' dorsal side of the left hand by a ring electrode. First, participants' skin was prepared with Nuprep Skin Preparation Gel and Ten20 Conductive Paste. Then a psychophysics procedure was carried out to establish the stimulation levels that would be used in the experiment. To do this, volunteers were asked to rate increasing levels of electrical stimulation on a scale of 0–10, with anchors at 3 (pain threshold) and 7 (highest tolerable pain to be repeatedly presented). The stimuli had a duration of 2 ms, and their intensity was increased by 1.25 mA from one stimulus to the next. This procedure was completed twice as done in previous behavioural studies (Delgado‐Sanchez et al. 2023; Hoskin et al. 2019), and the stimulations associated with ratings from 3 to 7 in the second completion were used in the cognitive task paradigm.

#### 2.2.2 Cued Pain Task

**FIGURE 1:**
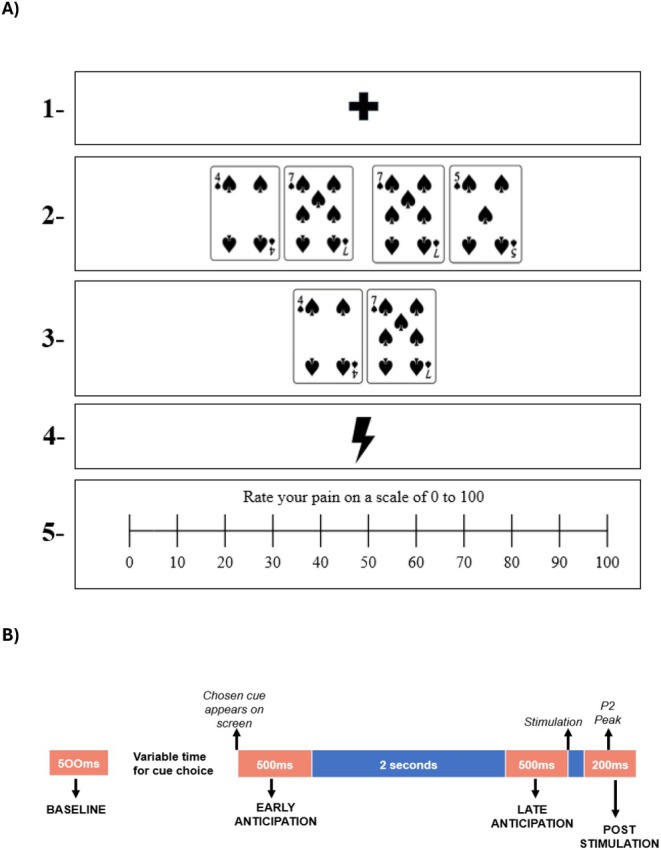
Trial structure and division of time windows of interest. (A) Trial structure as participants experienced it: (1) A fixation cross appears on the screen (duration: 0.5 s). (2) Participants are presented with two pairs of cards to choose from. In the figure, the pair on the left represents a 50% chance of getting pain 4 and a 50% chance of getting pain 7. The pair on the right represents a 50% chance of getting pain 7 and a 50% chance of getting pain 5. Participants were instructed to select the pair that led to the probability of less pain (in this case the left pair) (duration: variable, until choice is made). (3) The chosen pair is shown on the screen (duration: 3 s). (4) A stimulation corresponding to any of the values of the chosen pair is given to participants (duration: 2 ms). (5) A rating scale is shown on the screen and participants must rate the pain they have experienced (duration: variable, until choice is made). (B) Division of time windows of interest for analysis purposes. The time windows of interest are marked in red. Key events are presented above the timeline and the names given to the time windows below the timeline. The correspondence between the different sections of A and B is the following. Step 1 in A corresponds to ‘baseline’ in B; step 2 in A corresponds to ‘variable time for cue choice’ in B; step 3 in A corresponds to ‘early anticipation’, ‘late anticipation’ and the 2 s in between; step 4 in A corresponds to ‘stimulation’ and ‘post‐stimulation’ in B; step 5 does not have a corresponding time window in part 5.

The cognitive paradigm was presented with Psychtoolbox in MATLAB v2020a (Brainard 1997; Kleiner et al. 2007; Pelli 1997). Participants completed six practice trials plus 60 experimental trials. The trials were divided into six steps (Figure 1A). First, (1) a fixation cross was presented on the screen for 0.5 s. (2) Following that, two pairs of cards were presented on the screen, and participants were asked to pick the pair that led to the possibility of the lowest pain. The pair that participants were asked to pick was the target cue, and the one that they were asked to avoid was the lure cue. The target cue was composed of two numbers representing levels of pain stimulation rated during the psychophysics procedure. Each of the numbers had a 50% chance of being the level of stimulation delivered in the trial. The lure cue was also composed of two numbers representing possible levels of pain stimulation. In the case of the lure cue, one of the cards would show a number the same as one of the numbers represented in the target cue, and the other would always show a higher value than the alternative number in the target cue. Participants were trained to pick the target cue during the experiment instructions. During the practice trials, the experimenter gave feedback on performance. The goal of this decision task was to ensure the processing of the cue by participants, as well as to check their attention and understanding through the experiment. If a participant failed to select the target cue in over 25% of the trials, they were excluded from the analysis. The selection was performed by the participants by clicking on the left or right key of the mouse. The location of the target and lure cues on the screen (left or right) was randomised. The two cards composing the target cue always presented a difference of at least two units (e.g., 3 and 5) to ensure the independence of the estimation of the weight placed on stimulation and the cue. For a full discussion on this, see Delgado‐Sanchez et al. (2023). Participants were informed during the instructions that the level of stimulation delivered would be concordant with one of the numbers on the selected cue. Nevertheless, they were also informed that their experience of pain might change through the course of the experiment and that the goal was for them to rate the pain they had perceived in that specific trial. (3) After the cue selection, the selected cue (composed of two cards) was presented in the centre of the screen for 3 s. (4) The cards were removed from the screen, and a pain stimulation corresponding to one of the numbers shown on the target was delivered to participants. (5) One second after the stimulation, a rating scale from 0 to 100 was presented on the screen. This scale was chosen to be different from the initial 0–10 scale to decrease the fixation on the presented numbers and to aid in more variability in the responses as done in prior literature (Hoskin et al. 2019). Participants were asked to rate the pain they had experienced in that specific trial. (6) Finally, an inter‐trial interval of 1.5 s was set to avoid carryover effects from one trial to the next.

### 2.3 Psychological Questionnaires

After the task was completed, participants completed some validated psychological questionnaires.

#### 2.3.1 Five Facet Mindfulness Questionnaire

These 39‐item 5‐point Likert scales measure the mindfulness capacity of individuals and its impact on daily life. The scale presents five subscales corresponding to different factors within the construct of mindfulness: observing, describing, acting with awareness, non‐judging and non‐reacting (Baer et al. 2006).

#### 2.3.2 Experiences in Close Relationships‐Revised (ECR‐R)

This 7‐point Likert scale that presents 36 items measures adult attachment in a continuous manner through two dimensions: attachment anxiety and attachment avoidance (Fraley et al. 2000).

#### 2.3.3 Pain Catastrophizing Scale

This 5‐point Likert scale is formed by 13 items and measures the construct of pain catastrophising defined as ‘a set of exaggerated and ruminating negative cognitions and emotions during actual or perceived painful stimulation’ (Leung 2012, 204). The scale presents three subscales: magnification, rumination and helplessness (Sullivan et al. 1995).

### 2.4 Modelling of Pain Responses

**FIGURE 2:**
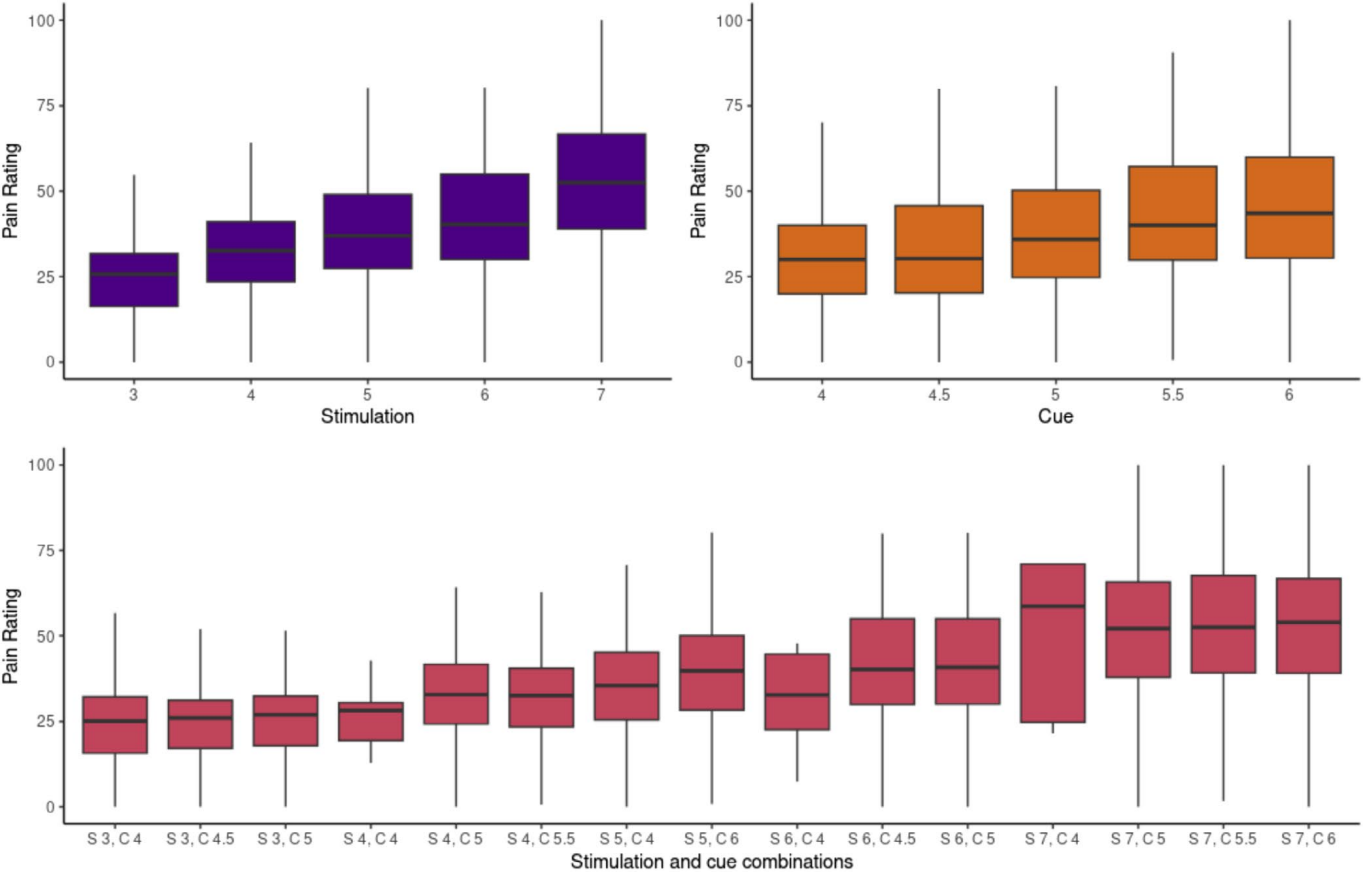
Pain ratings by stimulation, cue and stimulation + cue combinations. The figure above depicts the distribution of the pain ratings reported for each stimulation, cue and unique stimulation + cue combination across all participants.

Prior to the modelling, the values associated with stimulation levels and the cue mean (mean between the two presented cards) were re‐scaled to vary between 0 and 100. This was to ensure the stimulus intensity and cue values were on the same scale as the pain ratings. For descriptive purposes, Figure 2 shows the pain ratings associated with every stimulation level, cue mean, and stimulation and cue combinations.

In the present study, the participants' pain responses were modelled using the probabilistic framework proposed by Delgado‐Sanchez et al. (2023), which we briefly review here. This framework assumes that the participant (*j*) at trial (*i*) produces (infers) the pain rating (*Y*) by inverting a probabilistic generative model of the stimulus whereby both the stimulation/somatosensory input (*X*) and the observed average cue (*q*) are independently generated by the pain rating, with additional constraints representing a potential trait‐like bias. Mathematically, this probabilistic generative model can be written as the multiplication of three probability distributions (Equation 1), one for each factor considered. The first distribution models the observed stimulation as a Gaussian distribution around the pain rating value with participant‐specific precision parameter (${1/\beta }_{i}^{2}$) representing the weight placed on the stimulation. The second distribution models the observed average cue as a Gaussian distribution with participant‐specific precision parameter ($1/\rho^{2}$). The mean of this second distribution is modelled as the pain rating value plus an additive term, which is given by the standard deviation of the cue (SD) modulated by a participant‐specific scale parameter (η). Finally, the third distribution models the influence of the trait‐like bias as a Gaussian distribution on the pain ratings with trait‐like bias mean (μ) and the associated precision parameter ($1/\nu^{2}$) representing the weight placed on this bias. A summary of the meaning of all the parameters of the model is shown in Table 1.

(1) $$p\left(Y_{\mathrm{ij}}|X_{\mathrm{ij},}\beta_{i}^{2},q_{\mathrm{ij}},\rho_{i}^{2},{\mathrm{SD}}_{\mathrm{ij}},\eta_{i},\mu_{i},\nu_{i}^{2}\right)\propto p\left(X_{\mathrm{ij}}|Y_{\mathrm{ij},}\beta_{i}^{2}\right)\;p\left(q_{\mathrm{ij}}\;|Y_{\mathrm{ij},}\rho_{i}^{2},{\mathrm{SD}}_{\mathrm{ij}},\eta_{i}\right)\;p\left(Y_{\mathrm{ij}}|\mu_{i}{,\nu }_{i}^{2}\right)$$

**TABLE 1:** Parameter descriptions.

Parameter	Description
${1/\beta }_{i}^{2}$	Precision of the delivered stimulation distribution. Represents the level of certainty about the observed stimulus value. It is an indicator of the weight of sensory input on pain perception. Higher values indicate higher influence of somatosensory inputs.
$\eta_{i}$	A scale parameter representing the influence the standard deviation (SD) of the cue has on the representation of the cue. This parameter can take positive and negative values. A negative value indicates that a higher standard deviation is associated with a lower pain expectation; conversely, a positive value indicates that a higher standard deviation is associated with a higher pain expectation.
${1/\rho }_{i}^{2}$	Precision of the distribution of the observed average cue. Represents the level of certainty about the observed average cue value. Indicator of the weight of the observed cue on pain perception. Higher values indicate higher influence of the cue.
$\mu_{i}$	Mean of the trait‐like bias distribution. Indicator of the intrinsic bias of the participant's pain rating. Higher values indicate a tendency to give higher pain ratings irrespective of the observed cue and the delivered stimulation.
${1/\nu }_{i}^{2}$	Precision of the trait‐like bias distribution. Represent the certainty (strength) of the trait‐like bias. Indicator of the weight of the trait‐like bias on pain perception. Higher values indicate a higher reliance on the trait‐like bias, irrespective of the observed cue and the delivered stimulation.

Given the observed responses, the cues and stimuli input, the model's parameters were then inferred using Bayesian inference (model inversion) by means of the Hamiltonian Monte Carlo (HMC) algorithm implemented in the R package rstan (Stan Development Team 2022). To do this, the likelihood function in Equation (1) was first increased to incorporate independent prior distributions on all the parameters of the model. We refer the reader to our original paper (Delgado‐Sanchez et al. 2023) for more details on this procedure. We note that precision parameters were obtained by first estimating the associated variance parameters (*β* ^2^, *ρ* ^2^, *ν* ^2^) and then transforming them to represent precisions ($1/\beta^{2}$, $1/\rho^{2}$, $1/\nu^{2}$). This transformation facilitates interpretability and readability. While variance parameters represent the uncertainty (dispersion) about the factors, precisions represent the weight (certainty) placed on the factors.

### 2.5 EEG Data Collection and Pre‐Processing

In the following lines, the EEG data acquisition parameters are reported by the COBIDAS‐MEEG guidelines. Continuous EEG signals were recorded from 64 scalp electrodes from the BrainVision MR cap configured to the extended 10–20 system and a BrainAmp DC/MR amplifier. Impedance of electrodes was brought down to levels below 10 kohms. Participants were seated through the duration of the study. Pre‐processing of data was carried out with the EEGLAB toolbox (x2022.0) run in Matlab (vR2021a). The FCz electrode was used as a reference and AFz as the ground. The online sampling rate was 500 Hz. The pre‐processing steps included the application of a bandpass filter (0.1–40 Hz). The data were epoched from −4500 to +500 ms with respect to the stimulation. Visual inspection was conducted on the data and electrodes showing poor signal were interpolated and artefactual epochs were rejected. Poor signal was visually identified by the team. The median number of rejected trials was 3 (range 0–18), and the median number of interpolated electrodes was 1 (range 0–9). The median number of trials per participant for the analysis was 57 (range 42–60). Independent Component Analysis was then applied to identify and remove eye movements and muscle artefacts. The median number of rejected components was 2 (SD 2.36). Artefactual independent components were identified and rejected using the IClabel toolbox in EEGLAB. Participant trials were averaged to obtain participant ERPs.

Once epochs were considered artefact‐free, the trials were divided into four time windows (Figure 1B): Baseline, early anticipation, late anticipation and post‐stimulation. The baseline time window corresponded to 500 ms before the appearance of any cues on the screen, while participants were looking at the fixation cross. The early anticipation time window corresponded to the baseline‐corrected 500 ms after the target cue appearing on the screen (after selection). The late anticipation time window corresponded to the baseline‐corrected signal from 2500 to 3000 ms after the target cue appearing on the screen (after selection), which corresponds to the 500 ms window before the stimulation. Finally, the post‐stimulation time window was defined as the 100 ms before and after the ERP P2 peak of each participant. To do this, each participant's ERP was first calculated by averaging over trials in the 500 ms post‐stimulation, after correction with a baseline taken from the 500 ms prior to stimulation. The P2 peak was defined individually as the point of highest magnitude after stimulation. The electrode used to determine the latency and amplitude of the P2 peak was identified by selecting the electrode with the highest P2 amplitude after averaging the ERPs across participants. In the case of this study, the selected electrode was Cz.

SPM‐12 was used to perform EEG source reconstruction in each one of the time windows of interest. To do this, the template MRI image provided by SPM and the head shape information were used for co‐registration. After the computation of the forward model, a group inversion procedure was carried out to reconstruct the EEG sources. This procedure aggregates information across participants during the inversion, while the level of activation is still allowed to vary per participant. In this way, the group inversion improves generalisability (by mitigating the impact of individual variability and measurement noise), while accounting for subject‐specific effects. The following contrasts were applied to the source reconstruction images using a paired *t*‐test to identify the areas of activation‐deactivation in each time window: *baseline–early anticipation* (to identify the areas that are less active in early anticipation when compared to baseline), *early anticipation–baseline* (to identify the areas that are more active in early anticipation when compared to baseline), *baseline–late anticipation* (to identify the areas that are less active in late anticipation when compared to baseline), *late anticipation–baseline* (to identify the areas that are more active in late anticipation when compared to baseline), *late anticipation–post‐stimulation* (to identify the areas that are less active in post‐stimulation when compared to late anticipation) and *post‐stimulation–late anticipation* (to identify the areas that are more active in post‐stimulation when compared to late anticipation).

Statistical significance was assessed using peak level Family Wise Error (FWE) at *p* < 0.05. Once the anatomical areas (identified through the AAL3 atlas) that showed significant differences in each contrast were identified, the voxel with the greatest difference was used to create a spherical Region of Interest (ROI) with a 10 mm diameter. In some cases, the contrast analyses led to the identification of several significant clusters corresponding to the same anatomical area. In these cases, we created one ROI for each identified cluster as described above. Following this, a pair‐wise Spearman correlation analysis was performed between the averaged activities of the spheres representing the same brain structures. The ROIs with a correlation coefficient higher than 0.7 were considered to provide redundant information. This decision was based on findings from previous research showing that a correlation coefficient above 0.7 is indicative of concepts measuring the same constructs (Abma et al. 2016). If ROIs were found to provide redundant information, then the ROI closer to the centre of mass of the anatomical area was selected for further analysis. ROIs with correlation < 0.7 were all selected. The MarsBaR toolbox was used to extract the average activity of each ROI in the time window of interest.

### 2.6 Correlation Analyses

The correlation analyses between the model's parameters and the activity in each ROI were performed in R Studio. Violation of assumptions for parametric analysis was detected in most model parameters. Consequently, the follow‐up correlations were computed using non‐parametric Spearman correlation. In all subsequent tests, both hypothesised and exploratory analyses were carried out. The hypothesised analysis was done to test expected relationships. Correction for multiple comparisons was done by using the FDR method.

Due to limited research on the relative weight of each factor in pain perception, we lacked specific hypotheses regarding the expected relationship between some of the model parameters and some of the measured brain activity variables. In these cases, we tested for un‐hypothesised relationships through exploratory analysis. Note that due to the exploratory nature of this approach, the results reported here were not corrected for multiple comparisons and, therefore, they should be considered tentative findings to be confirmed by future research.

To conduct the hypothesised relationship analyses, we first identified which brain areas had been reported as relevant by prior research and came out as significant sources of activity in our contrast analyses. In the case of areas associated with sensory processing, prior research identified the somatosensory cortex, MFG, posterior cingulate cortex and inferior parietal cortex (Brown et al. 2008; Jones et al. 2010). In the case of areas associated with cue processing, prior research identified the subgenual cingulate cortex, MFG, superior frontal gyrus, inferior frontal gyrus, inferior temporal gyrus and orbitofrontal cortex as relevant areas (Brown et al. 2008; Watson et al. 2009). Consequently, we started by identifying which one of these areas surfaced as relevant in our contrast analyses and then ran a correlational analysis between activity in these areas and the respective weights. We hypothesised that we would see a positive correlation between the somatosensory weight and activity in areas previously associated with somatic processing, as well as a positive correlation between expectation weight and activity previously associated with cue processing. Finally, we also expected to see a positive correlation between activity previously associated with cue processing and the weight placed on uncertainty (*η*). Note that since the weight placed on uncertainty could have both positive and negative values (depending on whether it resulted in more positive or negative cue expectations, see Table 1 for more details), we used the absolute value of this parameter.

Since the contrast analyses identified more areas as relevant than those found in previous literature, we also conducted an exploratory correlational analysis between the model parameters and areas not identified as relevant by previous literature. Note that we did not have any hypotheses regarding the mean and weight of the trait‐like bias and the associated brain areas. Therefore, all analyses regarding these parameters were exploratory.

The second correlation analyses were carried out between the model parameters and the magnitude of the P2 peak of the scalp ERP. In the analysis for hypothesised relationships, we focussed on the correlation with the parameters $1/\beta^{2}$, η and μ. We hypothesised that a positive correlation would be found for the three parameters. This hypothesis was based on previous findings showing that the P2 peak correlated with the processing of sensory information (Nickel et al. 2022) (which could be related to the weight placed on stimulation/somatosensory input [$1/\beta^{2}$]), the uncertainty of the situation (Huang et al. 2017) (which could be related to the weight placed on uncertainty [η]) and the general perceived pain (Lee et al. 2009) (which could be related to the mean trait‐like bias in pain perception [μ]). We did not have any hypotheses regarding the correlation between the magnitude of the P2 peak and the parameters $1/\rho^{2}$ and $1/\nu^{2}$, consequently these were included in the exploratory analysis.

Finally, previous experimental studies have found positive correlation between attention to somatosensory input (a proxy of sensory weight; Trapp and Vilares 2020) and both analgesic (Johnston et al. 2012) and hyperalgesic (Buhle and Wager 2010) effects. Likewise, in clinical research, a higher attention to sensory information has also been linked to analgesic effects (e.g., mindfulness treatments; de Bruin et al. 2020) and hyperalgesic effects (e.g., chronic pain vulnerability due to hypervigilance; Vlaeyen and Linton 2000). These results raise the question of whether weight differences indicate protective or vulnerability factors regarding pain. To explore this issue further we conducted a third set of exploratory correlations to investigate the relationship between model parameters ($1/\beta^{2}$, η, $1/\rho^{2}$, μ, $1/\nu^{2}$) and psychological variables associated with pain protective (mindfulness) and vulnerability factors (attachment and pain catastrophising) (Harrison et al. 2019; Meredith et al. 2006; Sullivan et al. 2004).

## 3 Results

### 3.1 Source Reconstruction Results

**FIGURE 3:**
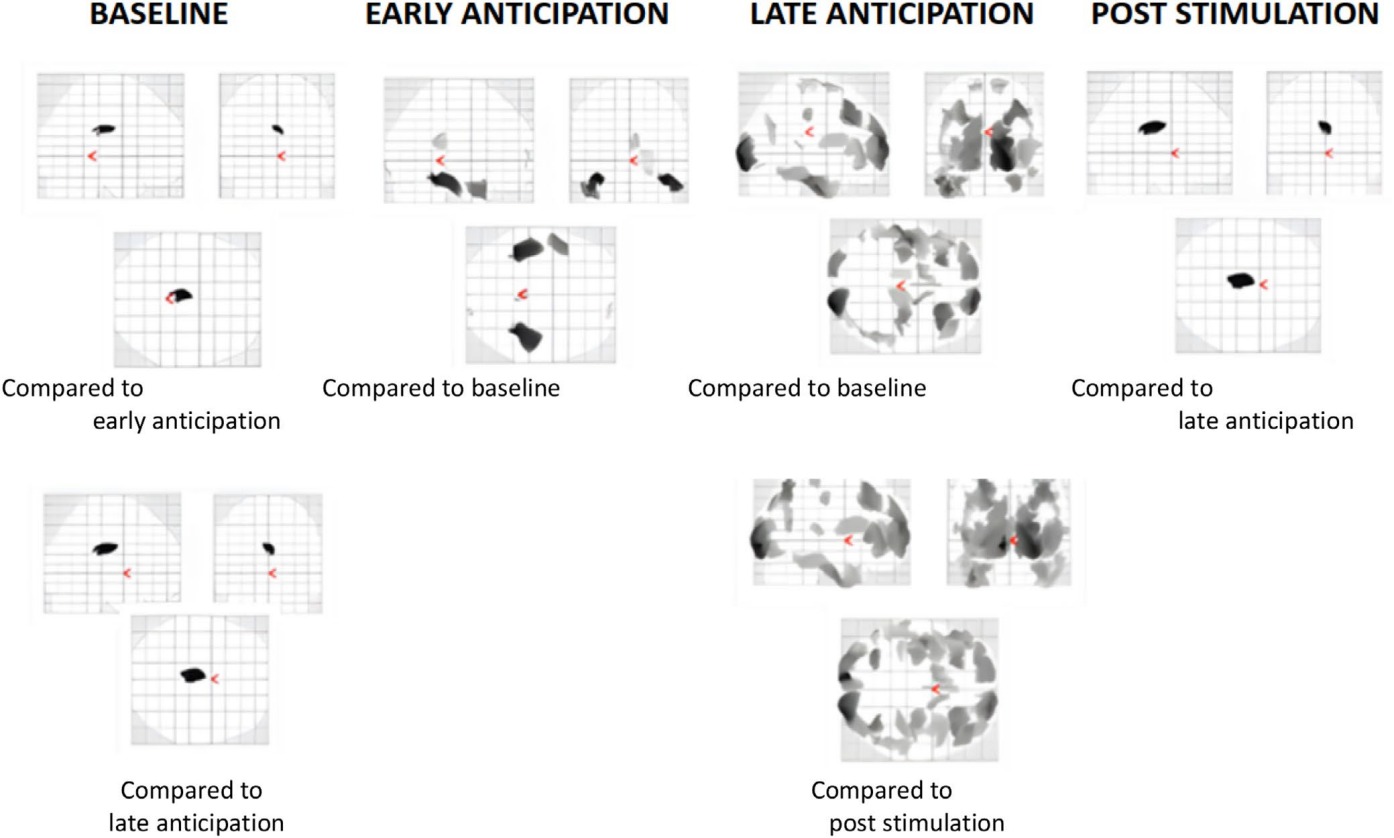
Glass brains representing the results of the contrast analyses. The figure displays areas of increased activity in each time window, relative to other time windows. Below each image, the specific time window used for comparison is specified.

Figure 3 shows the results of the contrasts. The early anticipation versus baseline contrast showed a reduction in activity in the cingulate cortex. Furthermore, an increase in activity in the following areas is observed: bilateral fusiform, left middle temporal gyrus, right lingual gyrus, right superior frontal gyrus and right inferior occipital gyrus.

In the late anticipation versus baseline contrast, a reduction in the cingulate cortex activity can be observed. Moreover, an increase in activity in the following areas can be observed: bilateral lingual, bilateral superior frontal gyrus, bilateral medial frontal gyrus, right inferior temporal gyrus, bilateral inferior frontal gyrus, right postcentral gyrus, right superior temporal gyrus, right parahippocampal cortex, left fusiform and the right precentral gyrus.

Finally, in the post‐stimulation versus late anticipation contrast, a reduction in activity in the following areas was observed: left calcarine, bilateral frontal medial gyrus, right angular, bilateral superior frontal gyrus, right inferior frontal gyrus, left middle occipital gyrus, left superior parietal cortex, bilateral medial temporal gyrus, right parahippocampal cortex, left fusiform, left caudate, bilateral postcentral gyrus, bilateral inferior temporal gyrus, right superior temporal gyrus. In addition, an increase in activity was observed in the midcingulate cortex.

In Supporting Information Table 2, the full results of the contrast analyses can be found with identified clusters and MNI coordinates.

#### 3.1.1 Source Reconstruction: Hypothesised Analyses

As previously mentioned, in the case of areas associated with sensory processing, prior research identified the somatosensory cortex, MFG, posterior cingulate cortex and inferior parietal cortex (Brown et al. 2008; Jones et al. 2010). Our contrast analyses showed two of these areas (MFG and somatosensory cortex) showed a statistically significant difference in activity in the late anticipation and post‐stimulation stages (in comparison to the other time windows they were compared to, which were the baseline and late anticipation time windows respectively).

**FIGURE 4:**
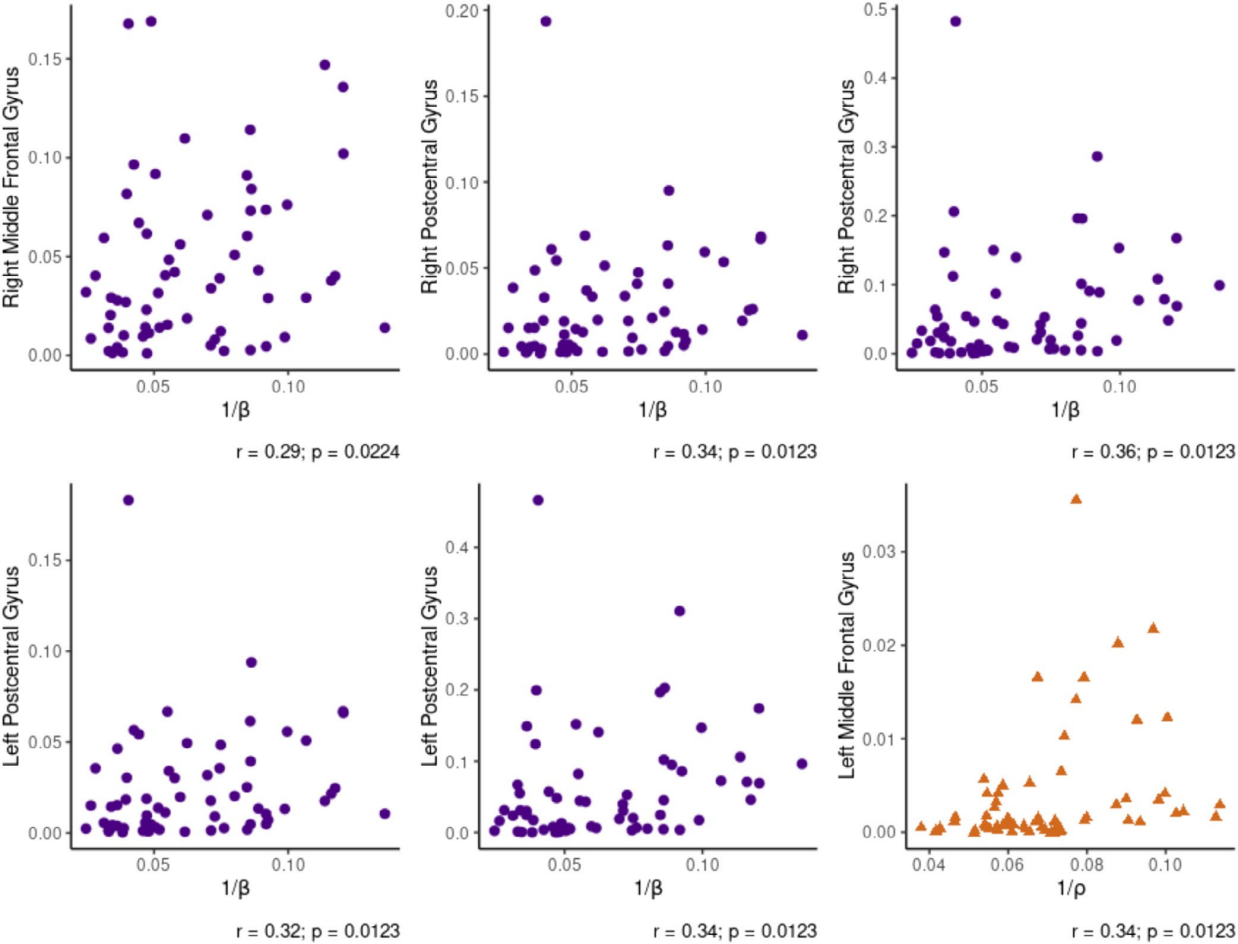
Relationship between model parameters and ROI activity. Scatterplots showing the relationship between mean source activity and estimated model parameters in brain regions and time windows where a significant correlation was found. Data from the late anticipation window are marked with purple circles; data from the post‐stimulation window with orange triangles.

Subsequent correlation results indicated a positive correlation between the input weight and activity in the right MFG and the bilateral postcentral gyrus during late anticipation. No significant correlations were found with activity in the post‐stimulation time window (Table 2, Figure 4).

**TABLE 2:** Spearman rho correlation between the weight placed on somatic input ${1/\beta }_{i}^{2}$ and activity at ROI (hypothesised analysis).

Brain structure	Right/Left	MNI (*x*, *y*, *z*)			Spearman rho
**Late anticipation**					
Middle frontal gyrus	R	42	22	44	0.29; corrected *p* = 0.0224*
	L	−26	26	34	0.22; corrected *p* = 0.0661
Postcentral gyrus	R	44	−22	38	0.34; corrected *p* = 0.0123*
	R	40	−36	60	0.36; corrected *p* = 0.0123*
	L	−42	−24	34	0.32; corrected *p* = 0.0124*
	L	−34	−38	62	0.34; corrected *p* = 0.0123*
**Post‐stimulation**					
Middle frontal gyrus	R	42	22	44	−0.03; corrected *p* = 0.760
	L	−28	26	48	−0.04; corrected *p* = 0.760
Postcentral gyrus	R	40	−36	60	−0.11; corrected *p* = 0.858
	L	−34	−38	62	−0.14; corrected *p* = 0.858

*Note:* This is a hypothesised analysis and has been subjected to FDR correction for multiple comparisons; the correction has been implemented at each time window.

*p < 0.05; **p < 0.01; ***p < 0.001.

In the case of areas associated with cue processing, prior research identified the subgenual cingulate cortex, MFG, superior frontal gyrus, inferior frontal gyrus, inferior temporal gyrus and orbitofrontal cortex as relevant areas (Brown et al. 2008; Watson et al. 2009). Of these, four areas showed significant source activity in our contrast analyses of the late anticipation and post‐stimulation time window, namely: superior frontal gyrus, MFG, inferior frontal gyrus and inferior temporal gyrus. Furthermore, the superior frontal gyrus also came out significant during the early anticipation period.

Results indicated a positive correlation between the weight placed on expectations and activity in the left MFG at the post‐stimulation time window. No activity in any other brain regions at any time showed a significant correlation with the parameter (Table 3, Figure 4). Furthermore, no significant correlation was found between the effect of the SD of the cue and activity in any ROI at any time window (Table 4).

**TABLE 3:** Spearman rho correlation between the weight placed on expectations ${1/\rho }_{i}^{2}$ and activity at ROI (hypothesised analysis).

Brain structure	Right/Left	MNI (*x*, *y*, *z*)			Spearman rho
**Early anticipation**					
Superior frontal gyrus	R	18	60	6	0.07; *p* = 0.569
**Late anticipation**					
Superior frontal gyrus	R	10	66	6	0.07; corrected *p* = 0.569
	L	−14	62	6	0.1; corrected *p* = 0.5518
Middle frontal gyrus	R	42	22	44	0.04; corrected *p* = 0.569
	L	−26	26	34	−0.04; corrected *p* = 0.676
	L	−36	54	10	0.01; corrected *p* = 0.588
Inferior frontal gyrus	R	40	38	0	−0.05; corrected *p* = 0.676
	L	−42	36	−16	0.05; corrected *p* = 0.569
Inferior temporal gyrus	R	50	−50	−22	−0.06; corrected *p* = 0.569
	L	−44	−10	−38	0.03; corrected *p* = 0.676
	L	−50	−34	−20	0.04; corrected *p* = 0.569
**Post‐stimulation**					
Superior frontal gyrus	R	10	66	6	0.2; corrected *p* = 0.338
Middle frontal gyrus	R	42	22	44	0.13; corrected *p* = 0.439
	L	−28	26	48	0.15; corrected *p* = 0.439
	L	−36	54	10	0.44; corrected *p* = 0.002*
Inferior frontal gyrus	R	40	30	0	0.22; corrected *p* = 0.338
Inferior temporal gyrus	R	50	−50	−22	0; corrected *p* = 0.588
	L	−50	−34	−20	0.15; corrected *p* = 0.439

*Note:* This is a hypothesised analysis and has been subjected to FDR correction for multiple comparisons; the correction has been implemented at each time window.

*p < 0.05; **p < 0.01; ***p < 0.001.

**TABLE 4:** Spearman rho correlation between the weight of the cue uncertainty $\eta_{i}$ and activity at ROI (hypothesised analysis).

Brain structure	Right/Left	MNI (*x*, *y*, *z*)			Spearman rho
**Early anticipation**					
Superior frontal gyrus	R	18	60	6	−0.08; *p* = 0.472
**Late anticipation**					
Superior frontal gyrus	R	10	66	6	−0.13; corrected *p* = 0.472
	L	−14	62	6	−0.07; corrected *p* = 0.540
Middle frontal gyrus	R	42	22	44	0.24; corrected *p* = 0.976
	L	−26	26	34	0.24; corrected *p* = 0.976
	L	−36	54	10	−0.04; corrected *p* = 0.730
Inferior frontal gyrus	R	40	38	0	0.06; corrected *p* = 0.939
	L	−42	36	−16	0.13; corrected *p* = 0.976
Inferior temporal gyrus	R	50	−50	−22	0.16; corrected *p* = 0.976
	L	−44	−10	−38	−0.12; corrected *p* = 0.540
	L	−50	−34	−20	0.1; corrected *p* = 0.976
**Post‐stimulation**					
Superior frontal gyrus	R	10	66	6	−0.15; corrected *p* = 0.472
Middle frontal gyrus	R	42	22	44	0.02; corrected *p* = 0.676
	L	−28	26	48	0.01; corrected *p* = 0.668
	L	−36	54	10	−0.25; corrected *p* = 0.472
Inferior frontal gyrus	R	40	30	0	−0.12; corrected *p* = 0.472
Inferior temporal gyrus	R	50	−50	−22	−0.1; corrected *p* = 0.486
	L	−50	−34	−20	−0.07; corrected *p* = 0.540

*Note:* This is a hypothesised analysis and has been subjected to FDR correction for multiple comparisons; the correction has been implemented at each time window.

#### 3.1.2 Source Reconstruction: Exploratory Analyses

In the contrast analyses applied to the EEG source data, a total of 44 clusters, corresponding to 18 brain structures, were identified (see Supporting Information for details). Not all these brain structures were predicted as relevant by our hypotheses. Furthermore, we had no specific hypotheses regarding the trait‐like bias and the weight placed on it. Consequently, the remaining potential correlations were explored to identify areas that might be of interest for future studies. The significant relationships encountered through this analysis can be found in Table 5.

**TABLE 5:** Summary of significant correlations not considered in study hypotheses (exploratory analysis).

Parameter	Brain structure	Right/Left	MNI (*x*, *y*, *z*)			Spearman rho
**Late anticipation**						
1/*β* ^2^	Inferior frontal gyrus	R	42	38	0	0.26; *p* = 0.036*
*η*	Postcentral gyrus	R	40	−36	60	0.26; *p* = 0.039*
*μ*	Middle frontal gyrus	R	42	22	44	0.26; *p* = 0.0411*
	Middle frontal gyrus	L	−26	26	34	0.26; *p* = 0.0408*
	Postcentral gyrus	R	44	−22	38	0.25; *p* = 0.0489*
		R	40	−36	60	0.29; *p* = 0.0219*
	Postcentral gyrus	L	−34	−38	62	0.26; *p* = 0.0384*
**Post‐stimulation**						
1/*ρ* ^2^	Middle temporal gyrus	R	−58	−6	−26	0.37; *p* = 0.0031**
		L	58	−2	−24	0.32; *p* = 0.0116*
1/*ν* ^2^	Middle frontal gyrus	L	−36	54	10	0.43; *p* = 0.0005***

*Note:* These are the results of exploratory analyses and therefore the significance values have not been corrected for multiple comparisons. 1/*β* ^2^: weight placed on somatosensory input; *η*: weight placed on the uncertainty of the cue; *μ*: trait‐like bias; 1/*ρ* ^2^: weight placed on the mean of the cue; 1/*ν* ^2^: weight placed on the trait‐like bias.

*p < 0.05; **p < 0.01; ***p < 0.001.

### 3.2 Correlation Between Model Parameters and the P2 Peak

**FIGURE 5:**
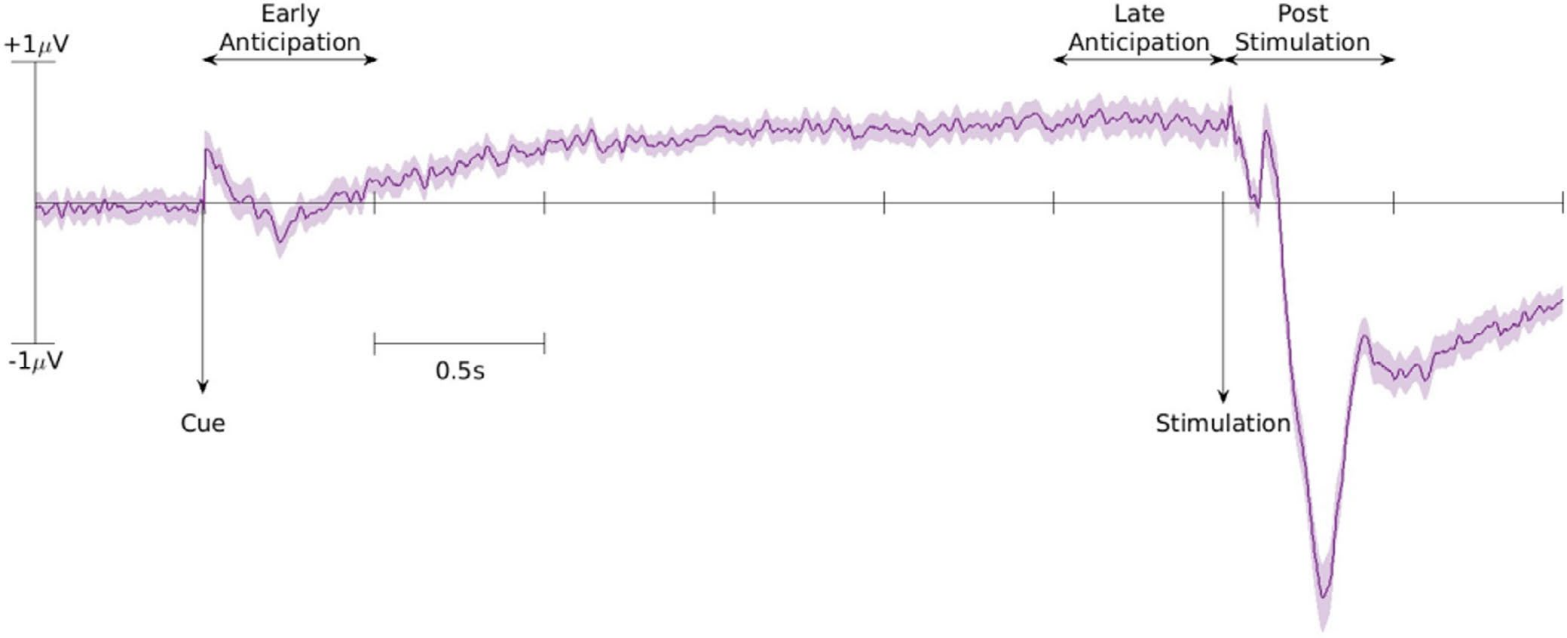
Grand average ERP with standard error across participants. The solid line represents the average ERP across all trials and all participants and the filled share around represents the standard error between participants. This ERP corresponds to electrode Fz. Note that the full post‐stimulation period was not included in our analysis. The P2 peak period was identified on each participants by identifying the time of the peak and then the data was epoched around this.

Regarding the hypothesised effect analyses, as expected, results showed medium significant positive correlations between the P2 magnitude and the values of $1/\beta^{2}$ (0.27; *p* = 0.0349), η (0.28; *p* = 0.0349) and μ (0.30; *p* = 0.0349) (Table 6). In the exploratory analyses no significant correlations were observed between the P2 peak and the $1/\rho^{2}$ and $1/\nu^{2}$ parameters (Table 7). In Figure 5, the full ERP graph is presented.

**TABLE 6:** Spearman rho correlation of sensory weight ${1/\beta }_{i}^{2}$, the uncertainty of the cue $\eta_{i}$ and trait‐like $\mu_{i}$ with the amplitude of the P2 peak.

	${1/\beta }_{i}^{2}$	$\eta_{i}$	$\mu_{i}$
P2 peak amplitude	0.27; corrected *p* = 0.0349	0.28; corrected *p* = 0.0349	0.30; corrected *p* = 0.0349*

*Note:* This is a hypothesised analysis and has been subjected to FDR correction for multiple comparisons.

*p < 0.05; **p < 0.01; ***p < 0.001.

**TABLE 7:** Spearman rho correlation of the weights placed on the cue ${1/\rho }_{i}^{2}$ and trait‐like bias ${1/\nu }_{i}^{2}$ with the amplitude of the P2 peak.

	${1/\rho }_{i}^{2}$	${1/\nu }_{i}^{2}$
P2 peak amplitude	0.10; *p* = 0.4452	−0.08; *p* = 0.5084

*Note:* This is an exploratory analysis and has not been subjected to FDR correction for multiple comparisons.

### 3.3 Model Parameters and Psychological Variables

No significant correlations were found between the model parameters and pain catastrophising, attachment or mindfulness. However, the mean of the pain ratings showed to be significantly correlated with all model parameters. A full summary of the results can be found in Table 8.

**TABLE 8:** Spearman rho correlation of model parameters with psychological variables (exploratory analysis).

	Mindfulness	Attachment anxiety	Attachment avoidance	Pain catastrophising	Mean of pain ratings
${1/\beta }_{i}^{2}$	−0.06; *p* = 0.66	0.12; *p* = 0.33	0.12; *p* = 0.34	−0.01; *p* = 0.94	0.79; *p* < 0.001***
${1/\rho }_{i}^{2}$	−0.12; *p* = 0.36	0.00; *p* = 0.98	0.08; *p* = 0.53	0.08; *p* = 0.55	0.31; *p* = 0.0123*
$\eta_{i}$	0.00; *p* = 0.98	0.12; *p* = 0.35	−0.01; *p* = 0.95	−0.03; *p* = 0.81	0.97; *p* < 0.001***
$\mu_{i}$	−0.06; *p* = 0.65	0.13; *p* = 0.62	−0.03; *p* = 0.84	−0.01; *p* = 0.95	0.99; *p* < 0.001***
1/*ν* ^2^	0.02; *p* = 0.88	−0.09; *p* = 0.47	0.08; *p* = 0.52	0.04; *p* = 0.77	−0.47; *p* < 0.001***

*Note:* This is an exploratory analysis and has not been subjected to FDR correction for multiple comparisons.

*p < 0.05; **p < 0.01; ***p < 0.001.

## 4 Discussion

This study aimed to explore the EEG correlates of the weight placed on somatosensory input, expectations and trait‐like bias during pain perception. Understanding weights as the quantitative weights assigned to each of the factors influencing pain perception (somatic input, expectations and trait‐like bias) during the process of pain perception, as derived from Bayesian computational models. To meet the aims of the study, we tested the correlations of these weights with the main sources of brain activity during a cued pain task. Analyses on hypothesised effects showed that the weight placed on somatosensory input was associated with higher activity in areas associated with attention (MFG) and somatosensory processing (postcentral gyrus) during the late anticipation of pain. Whereas the weight placed on the cue was associated with higher activity in areas related to attention (medial frontal gyrus) during the pain perception stage (post‐stimulation). Exploratory analyses also showed a correlation between the weight placed on the cue and activity in semantic processing areas (medial temporal gyrus) during pain perception. Furthermore, the correlation analysis of these weights with pain intensity and pain protective (mindfulness) and vulnerability (pain catastrophising and attachment insecurity) showed that both the weight placed on somatosensory input and expectations were associated with the perception of higher pain intensity, whereas the weight placed on the trait‐like bias was associated with lower mean pain intensity. There was no correlation between any of the weights and any of the other measured psychological variables.

Regarding the source activity data, our results indicate that the weight placed on somatosensory input is correlated with activity in the MFG and the postcentral gyrus during the late anticipation stage. These results are concordant with the findings of previous research (Brown et al. 2008; Jones et al. 2010) providing some validation of our results. Interestingly, activity in the MFG has been associated with the top‐down regulation of attention to task‐relevant information (Ciaramelli et al. 2010; Comte et al. 2016). Therefore, the increased activity in this area during the late anticipation stage, and the concomitant activity of the postcentral gyrus and the inferior frontal gyrus (which have been associated with interoceptive processing; Du et al. 2023; Terasawa et al. 2011) indicate that the sensory weight could be related to the level of attention directed to sensory information after cue processing.

Regarding the weight placed on the cue, the neural correlates show that a higher weight on expectations/cue is associated with greater activity in the MFG after pain stimulation. Firstly, this serves to validate the results of the model since prior research exploring the placebo effect has found activity in this area to be correlated to a higher influence of expectations (Watson et al. 2009). Moreover, the activity in the MFG indicates once again that the weight could be representing an attentional process. When it comes to the concomitant activity that could serve to identify the target of attention, our exploratory analyses identified that the expectation weight is also correlated with activity in the middle temporal gyrus (an area associated with semantic processing; Davey et al. 2016). Furthermore, it is interesting to note that activity in the MFG has also been associated with context updating (D'Ardenne et al. 2012), which has been proposed to start taking place at the same time as the P2 peak (Lenartowicz et al. 2010). Consequently, activity in this area during the post‐stimulation period could also be indicating a context updating process and not just an attentional process. In this way, participants who are more influenced by the cue could be those who compare to a greater extent the received pain stimulation with the presented cues and update their internal model accordingly.

All in all, the correlation of the different weights with activity in the MFG provides evidence for the idea that the weight placed on a factor is representative of the attention directed to it, as proposed by previous research (Trapp and Vilares 2020). This way, the ability to switch attention to somatosensory information after being presented with cues seems to increase the weight placed on somatosensory input. Alternatively, switching attention to priorly received information after receiving stimulation seems to increase the weight placed on expectations.

Before finishing with the interpretation of the neuroimaging evidence, the pattern of activation from baseline to early anticipation, late anticipation and stimulation can also give us some information about the general behaviour of our sample. At baseline, participants show a higher activation of the midcingulate cortex, which has been linked to attention towards pain stimuli (Brown and Jones 2008) and fear and avoidance (Vogt 2005). This could indicate that trials might start with fear about the upcoming experience. Once the cue is presented, activity in these areas decreases, whilst activity in areas related to cue processing (visual processing) increases. As the anticipation period advances and the late anticipation period is reached, activity in areas associated with somatosensory processing and attentional regulation increases. This pattern could indicate that when the cue is first presented, cognitive resources are focused on its interpretation. Nevertheless, as more time is given to participants and, therefore, the analysis of the cue is possibly completed, participants switch to focus their attention on the upcoming stimulation. Once stimulation is received, participants reduce activity in sensory and attentional brain areas, and activity in the midcingulate cortex increases, similar to baseline. In this process, it is particularly interesting that as anticipation advances, the areas associated with somatosensory processing and attention show increased activity. This finding raises the question of whether the anticipation time would influence the weight placed on the different components. Prior research has already encountered that an increase in the duration of anticipation increases pain ratings (Clark et al. 2008; Hauck et al. 2007), consequently, future research could explore if this increase is due to an increase in the weight placed on somatosensory input. This could be carried out by manipulating anticipation time and observing how it changes the weight placed on the different factors.

It is interesting to note that both the weight placed on the somatosensory input and the cue are associated with hyperalgesic effects. This is concordant with prior research that encountered a higher perceived pain intensity when attention was focused on pain stimulation (Bukola and Paula 2017; Rischer et al. 2020). This study corroborates this result and also provides evidence that a higher weight on pain‐related cues (and not only somatosensory input) also leads to hyperalgesic effects. Nevertheless, our results are contradictory to some other evidence pointing at an analgesic effect of attention to sensory information (Johnston et al. 2012). One potential explanation for the discrepancy with this study could rely on the nature of the employed task. In the Johnston et al. (2012) study, they induced sensory focus by asking participants to focus their attention on the descriptive component of a heat detection task. They found that this led to analgesic effects, especially in cases in which the cues provided to participants indicated high‐intensity upcoming pain. Since our results also indicate that focusing attention on pain‐related cues leads to hyperalgesia, it could be argued that the participants in the Johnston et al. (2012) study might have experienced analgesia through the distraction from pain‐related cues and the fixation on the descriptive aspects of heat.

There are certain limitations of this study. To begin with, we used a template MRI to conduct the source reconstruction. For this reason, we took certain measures to limit spatial resolution issues (such as performing a group inversion). Our results were in line with our hypotheses, and often several clusters were identified for the same structures, giving some reassurance in regard to the validity of our categorisations. Nevertheless, future studies could aim to improve spatial resolution by using individual MRIs in the source reconstruction process. Alternatively, fMRI could also be used as a way to improve spatial resolution; however, since our results show that the correlation between the parameters and brain activity is time dependent, the high temporal resolution of EEG might still render it the best technique to keep on exploring this topic. A balanced exploration of both techniques should be carried out in subsequent investigations.

Another important limitation of our study is the sample used. This experiment was conducted in a group of healthy participants, which is a positive factor for limiting confounding variables; however, it does pose the issue of a very homogeneous sample. The consequences of this could be affecting our results. For instance, we could be having a lower variability in our Bayesian estimates than we would find in the general population. Consequently, this study should be replicated in chronic pain populations.

Moreover, future research should also focus on answering some of the questions generated by this study. This work was used to provide some preliminary construct validity for the measured parameters without relying on psychological variables, which often carry an inherent valence. For instance, attempting to validate the weight placed on somatosensory input using psychological variables like mindfulness (positive valence) or hypervigilance (negative valence) could complicate interpretation due to the complexity and emotional biases of these variables. By using EEG, we demonstrate that the weight placed on stimulation is linked to somatic processing independent of any valence. Additionally, we found that both the weight placed on stimulation and on the cue exhibit hyperalgesic effects. Therefore, future research should explore how these constructs relate to psychological variables with negative valence, such as hypervigilance. Other promising directions include investigating correlations between different parameters, examining neural correlates of combined parameter profiles (e.g., individuals with high reliance on somatic input but low reliance on cues), and exploring how these effects vary across different time windows. Finally, as mentioned previously, investigations should attempt to confirm the effects on the different weights of the brain areas identified through exploratory analyses. Furthermore, regarding the behavioural results, whether the analgesic/hyperalgesic effects of sensory weight depend on the presented cues and their associated weight should be studied.

In conclusion, the study has ascertained initial concurrent validity, via EEG correlates, for Bayesian estimates of sensory and expectation weights. In addition, the EEG sources identified are consistent with the interpretation that the weight placed on each factor is related to the degree of attention to the relevant sources of information, which can occur at different times during the pain anticipation–perception process. All in all, these results indicate that the different weight estimates might be valid measures to be used for phenotyping individuals' pain responses and could therefore be a useful resource for personalised medicine.

## Author Contributions

This study was designed by A.D.‐S., C.B., A.J. and H.S. The experiments were performed by A.D.‐S. The data were analysed by A.D.‐S. under the supervision and advice of C.B., N.J.T.‐B. and C.C., and the results were critically examined by all authors. A.D.‐S. had a primary role in preparing the manuscript, which was edited by all co‐authors. All authors have approved the final version of the manuscript and agree to be accountable for all aspects of the work.

## Conflicts of Interest

The authors declare no conflicts of interest.

## Supporting information


**Tables S1–S6:** ejp70154‐sup‐0001‐TablesS1‐S6.docx.

## Data Availability

The data supporting the findings reported in this paper are openly available at https://osf.io/4kgtp/?view_only=06ad948e0b8e4d9bbc60032d4f9a223.
