# Supplementary material for: EEG Correlates of the Influence of Somatosensory Input, Expectations and Trait‐Like Bias on Pain Perception
Source: Eur J Pain. 2025 Nov 7;29(10):e70154. doi: 10.1002/ejp.70154 (PMC12594617; doi:10.1002/ejp.70154)
Supplement: Supplementary file 1 — Tables S1–S6: ejp70154‐sup‐0001‐TablesS1‐S6.docx. [file EJP-29-0-s001.docx]

Supplementary materials

Supplementary materials 1: Results of contrast analyses

Supplementary table 1. Results of contrast Baseline > Early anticipation

| **Cluster number** | **Volume (number of voxels)** | **X** | **Y** | **Z** | **Label** |
| --- | --- | --- | --- | --- | --- |
| 1 | 262 | -2 | -20 | 26 | Midcingulate cortex (left) |
|  |  | -4 | -34 | 24 | Posterior cingulate cortex (left) |

Supplementary table 2. Results of contrast Early anticipation > Baseline

| **Cluster number** | **Volume (number of voxels)** | **X** | **Y** | **Z** | **Label** |
| --- | --- | --- | --- | --- | --- |
| 1 | 605 | 36 | -50 | -18 | Fusiform (right) |
| 2 | 712 | -36 | -52 | -20 | Fusiform (left) |
|  |  | -44 | -48 | -20 | Inferior Temporal Gyrus (left) |
|  |  | -40 | -68 | -18 | Fusiform (left) |
| 3 | 405 | -48 | 6 | -32 | Middle Temporal Gyrus (left) |
|  |  | -54 | 0 | -30 | Middle Temporal Gyrus (left) |
| 4 | 179 | 22 | -94 | -16 | Lingual gyrus (right) |
|  |  | 12 | -92 | -8 | Lingual gyrus (right) |
|  |  | 10 | -96 | 2 | Calcarine sulcus (right) |
| 5 | 255 | 18 | 60 | 6 | Superior frontal gyrus (right) |
|  |  | 26 | 62 | 4 | Superior frontal gyrus (right) |
|  |  | 12 | 62 | -6 | Fronto medial orbital cortex (right) |
| 6 | 140 | 44 | -70 | -14 | Inferior Occipital Gyrus (right) |
|  |  | 54 | -62 | -8 | Inferior Temporal Gyrus (right) |

Supplementary table 3. Results of contrast Baseline > Late anticipation

| **Cluster number** | **Volume (number of voxels)** | **X** | **Y** | **Z** | **Label** |
| --- | --- | --- | --- | --- | --- |
| 1 | 260 | 0 | -24 | 24 | Midcingulate cortex (bilareral) |
|  |  | -2 | -34 | 24 | Posterior cingulate cortex (left) |

Supplementary table 4. Results of contrast Late anticipation > Baseline

| **Cluster number** | **Volume (number of voxels)** | **X** | **Y** | **Z** | **Label** |
| --- | --- | --- | --- | --- | --- |
| 1 | 755 | 22 | -100 | -8 | Lingual gyrus (right) |
|  |  | 16 | -102 | 2 | Calcarine sulcus (right) |
|  |  | 12 | -98 | 8 | Cuneus (right) |
| 2 | 817 | 14 | 62 | 8 | Superior frontal gyrus (right) |
|  |  | 8 | 64 | -6 | Frontal medial orbital gyrus (right) |
|  |  | 18 | 60 | 20 | Superior frontal gyrus (right) |
| 3 | 421 | -12 | -98 | -8 | Calcarine sulcus (left) |
|  |  | -18 | -100 | -14 | Lingual gyrus (left) |
|  |  | -22 | -102 | 2 | Middle Occipital gyrus (left) |
| 4 | 928 | -14 | 62 | 6 | Superior frontal gyrus (left) |
|  |  | -16 | 54 | 26 | Superior frontal gyrus (left) |
|  |  | -8 | 62 | 16 | Superior frontal gyrus (left) |
| 5 | 375 | 24 | 22 | 34 | Superior frontal gyrus (right) |
|  |  | 36 | 22 | 48 | Medial frontal gyrus (right) |
|  |  | 44 | 24 | 38 | Medial frontal gyrus (right) |
| 6 | 452 | -44 | -10 | -38 | Inferior Temporal Gyrus (right) |
|  |  | -58 | -26 | -30 | Inferior Temporal Gyrus (right) |
|  |  | -52 | -20 | -26 | Inferior Temporal Gyrus (right) |
| 7 | 247 | -26 | 26 | 34 | Medial frontal gyrus (left) |
|  |  | -34 | 26 | 36 | Medial frontal gyrus (left) |
|  |  | -26 | 20 | 44 | Medial frontal gyrus (left) |
| 8 | 300 | 42 | 38 | 0 | Inferior Frontal gyrus (pars triangularis) (right) |
|  |  | 42 | 40 | -8 | Inferior Frontal gyrus (pars orbitalis)(right) |
|  |  | 48 | 30 | 12 | Inferior Frontal gyrus (pars triangularis) (right) |
| 9 | 257 | -42 | 36 | -16 | Inferior Frontal gyrus (pars orbitalis)(left) |
|  |  | -52 | 22 | 0 | Inferior Frontal gyrus (pars triangularis) (left) |
|  |  | -40 | 40 | -2 | Inferior Frontal gyrus (pars triangularis) (left) |
| 10 | 42 | 44 | -22 | 38 | Postcentral gyrus (right) |
| 11 | 50 | 58 | -34 | 8 | Superior Temporal gyrus (right) |
| 12 | 79 | 32 | -20 | -28 | Parahippocampal cortex (right) |
| 13 | 65 | -34 | -20 | -32 | Fusiform (left) |
| 14 | 63 | 50 | -50 | -22 | Inferior Temporal gyrus (right) |
|  |  | 52 | -42 | -18 | Inferior Temporal gyrus (right) |
| 15 | 99 | 14 | -28 | 62 | Precentral gyrus (right) |
|  |  | 14 | -28 | 68 | Precentral gyrus (right) |

Supplementary table 5. Results of contrast Late anticipation > Stimulation

| **Cluster number** | **Volume (number of voxels)** | **X** | **Y** | **Z** | **Label** |
| --- | --- | --- | --- | --- | --- |
| 1 | 2293 | -10 | -96 | -8 | Calcarine sulcus (left) |
|  |  | 24 | -98 | -10 | Lingual gyrus (right) |
|  |  | -8 | -102 | 0 | Calcarine sulcus (left) |
| 2 | 462 | 42 | 22 | 44 | Frontal medial gyrus (right) |
|  |  | 24 | 22 | 34 | Superior frontal gyrus(right) |
| 3 | 130 | 38 | -66 | 48 | Angular (right) |
|  |  | 36 | -64 | 40 | Angular (right) |
|  |  | 36 | -76 | 36 | Middle occipital gyrus (right) |
| 4 | 823 | 10 | 66 | 6 | Superior frontal medial gyrus (right) |
|  |  | 20 | 56 | 22 | Superior frontal gyrus (right) |
|  |  | 8 | 64 | -6 | Frontal medial orbital gyrus (right) |
| 5 | 433 | -28 | 26 | 48 | Frontal medial gyrus (left) |
|  |  | -26 | 26 | 34 | Superior Frontal gyrus (left) |
|  |  | -34 | 26 | 36 | Medial frontal gyrus (left) |
| 6 | 669 | 40 | 38 | 0 | Inferior Frontal gyrus (pars triangularis) (right) |
|  |  | 52 | 26 | 0 | Inferior Frontal gyrus (pars triangularis) (right) |
|  |  | 48 | 30 | 12 | Inferior Frontal gyrus (pars triangularis) (right) |
| 7 | 1397 | -36 | 54 | 10 | Medial frontal gyrus (left) |
|  |  | -30 | 46 | -2 | Superior frontal gyrus (left) |
|  |  | -14 | 54 | 28 | Superior frontal gyrus (left) |
| 8 | 61 | -32 | -80 | 38 | Middle occipital gyrus (left) |
|  |  | -32 | -84 | 30 | Middle occipital gyrus (left) |
| 9 | 79 | -32 | -68 | 50 | Superior parietal gyrus (left) |
| 10 | 152 | -58 | -6 | -26 | Middle temporal gyrus (left) |
|  |  | -50 | -4 | -24 | Middle temporal gyrus (left) |
|  |  | -50 | 4 | -34 | Middle temporal gyrus (left) |
| 11 | 100 | 58 | -2 | -24 | Middle temporal gyrus (right) |
|  |  | 62 | -12 | -20 | Middle temporal gyrus (right) |
| 12 | 132 | 32 | -18 | -30 | Parahippocampal cortex (right) |
| 13 | 121 | -34 | -18 | -34 | Fusiform (left) |
| 14 | 198 | 0 | 12 | 12 | Cuadate (left) |
| 15 | 48 | -42 | -24 | 34 | Postcentral gyrus (left) |
|  |  | -48 | -22 | 40 | Postcentral gyrus (left) |
| 16 | 49 | 44 | -22 | 38 | Postcentral gyrus (right) |
| 17 | 154 | 40 | -36 | 60 | Postcentral gyrus (right) |
|  |  | 34 | -34 | 54 | Postcentral gyrus (left) |
| 18 | 82 | 50 | -50 | -22 | Inferior Temporal gyrus (right) |
|  |  | 52 | -42 | -18 | Inferior Temporal gyrus (right) |
| 19 | 145 | -34 | -38 | 62 | Postcentral gyrus (left) |
| 20 | 44 | 56 | -32 | 6 | Superior Temporal gyrus(right) |
| 21 | 192 | -50 | -34 | -20 | Inferior Temporal gyrus (left) |
|  |  | -52 | -22 | -22 | Inferior Temporal gyrus (right) |

Supplementary table 6. Results of contrast Stimulation > Late anticipation

| **Cluster number** | **Volume (number of voxels)** | **X** | **Y** | **Z** | **Label** |
| --- | --- | --- | --- | --- | --- |
| 1 | 298 | -6 | -26 | 26 | Midcingulate cortex (left) |
|  |  | -4 | -34 | 24 | Posterior cingulate cortex (left) |
